# The Odontological Society of Great Britain

**Published:** 1885-07

**Authors:** 


ARTICLE III.

### THE ODONTOLOGICAL SOCIETY OF GREAT BRITAIN.

The last ordinary Monthly Meeting of the above Society for the present Session was held at 40, Leicester Square, on Monday evening, June 1st, Mr. C. Spence-Bate, F. R. S., President, in the Chair.

Mr. Oakley Coles, in the absence of the Librarian, announced the receipt, as an addition to the library, of Dr. Herbst's work on "Filling Teeth with Gold by the New Method."

Mr. Redman remarked, with regard to a case that he showed of absorption of a first molar consequent upon the pressure of the bicuspid, that the tooth which had caused the absorption was so low down that the crown could hardly be seen, and at the time of consultation it was mere conjecture that the pressure of that tooth might be the cause of the pain the child was suffering from. The patient was fourteen years of age, and the amount of absorption in the tooth was very surprising.

He also showed a split bicuspid. There was nothing in any way to account for the fracture, and it must consequently have been from "explosion of the pulp."

The President showed the model of a case of a split second molar which he had treated, first with elastic bands and afterwards with a little gold wire tied round to bring the parts together. He had then treated the tooth with chloroform, drilled it out, and filled with amalgam. The tooth gave every sign of being once again very useful.

Communications were also made by Mr. Andrew Wilson, Mr. Maggs, and Mr. Coles.

Mr. C. S. Tomes made a communication on

### SOME EXPERIMENTS ON AMALGAM FILLINGS.

He said that he felt almost ashamed to bring before the Society the very small number of experiments he had made lately with amalgams, especially when he considered the very careful and extended tests which had been made by Dr. W. St. George Elliott. The experiments he wished to mention were very few in number, but inasmuch as they were of a different kind from those made by Dr. Elliott, and were all of a practical tendency, he thought it well to place them before the Society that evening, especially as it would be some time before he should be able to continue his experiments.

The direction he had taken up was the question of water-tight fillings made by amalgams. He had only used plastic amalgams in his experiments, as he considered that they wanted an amalgam which was sufficiently plastic to be easy to work. This was not the case with a dry amalgam. The only thing which had induced him to make the experiment he had done was that Dr. Bonwill had showed him at Philadelphia, last Christmas, some amalgam fillings of exceptional excellence, and had also exhibited his method, which he (Mr. Tomes) considered to be quite different from what he had before seen, it being, in fact, virtually to squeeze the amalgam into the cavity of the tooth. Dr. Bonwill's method was to put the amalgam into the cavity in a very plastic form, make a pad of amadou or paper and so squeeze the amalgam into the cavity, removing from time to time the more plastic portions of the amalgam which would squeeze out of the cavity. In certain cavities Dr. Bonwill used the adjoining tooth as a matrix; and in cases of the cavities in two teeth meeting, Dr. Bonwill filled the space as one, and then cuts through the amalgam between the two cavities.

Mr. C. S. Tomes said the great difference between the usual method and that employed by Dr. Bonwill was, that the mercury from the amalgam was practically squeezed out from the cavity, and not done before inserting the plug. He (Mr. Tomes) had in using amalgams discarded all varnishes. If the stopping was water-tight in a cavity the most penetrating ink stain could not get in. He had heard it said by some that Draper's ink was too strong a test, but he did not consider that such was the case. If a plug were really water-tight the ink could not stain it; if it did stain, it was quite evident that contraction in setting was the cause, or that it could never have been a water-tight filling.

He had found that if he took some Bonwill's amalgam, mixed in a tolerably plastic form, and then packed it into the cavity with burnishers, he did not get a water-tight plug.

In the experiments which had been carried out by Mr. Baldwin and himself, they had both found that if they plugged a tooth by Dr. Bonwill's method, they could depend upon their plug being water-tight.

This applied also to several other amalgams which they had experimented with in addition to Dr. Bonwill's.

If at any time they had an amalgam filling which was not water-tight, the edge should always be looked to, as he had found that in cases where the cavity had a thin feather edge, leakage was invariably the result. This seemed to be a very important point with regard to a cavity for an amalgam filling (it was of course the reverse for gold fillings.) It was unfortunately in some cases unavoidable, as feather-edged cavities would occur; but they could depend upon one fact, and that was, the squarer the edge of their cavity, the more water-tight would be their plug.

Packing amalgam with burnishers, not only with the Bonwill amalgam, but also in the standard alloy, was not satisfactory; they could only get water-tight fillings more or less, but no dependence could be placed upon them.

He also tried putting into the plastic amalgam pieces of amalgam which had previously become set, so as to lessen the shrinkage of the setting mass. He had also tried using a piece of amalgam (previously set) shaped to fit the cavity, and then carefully packed all round in a very solid manner. All the various experiments he had adopted, as far as Mr. Baldwin and himself had experimented, they were led to the conclusion that Bonwill's method was the best, namely, to put the material in, in a very plastic condition, pack it with heavy pressure, and remove the soft portions as they came out of the cavity.

Mr. Tomes, in conclusion, said that his experiments had not been numerous enough or sufficiently widely extended to prove anything, and must be simply regarded as suggestions.

They wanted to use a good plastic amalgam in large molar crown cavities, and it would be a good thing if they could find out the best method of manipulating it. Dr. Bonwill's method of packing amalgam was described as follows by Mr. Ewbank in the *Journal of the British Dental Association*:—

"I wish to communicate to the profession a rapid, easy and effective method of plugging teeth with amalgam, kindly shown to me by its originator, Mr. Bonwill, of Philadelphia; and as this way is particularly suitable for contour work, I will describe the filling of two interstitial cavities, taking for example the proximal surfaces of a first and second molar. The teeth having been previously separated, the cavities are excavated, the cervical margins, when practicable, being cut down to the level of the gum, and the lingual and labial walls largely removed, so that the edges of the fillings may be well exposed. Small grooves are then cut on either side, and, as a rule, retaining cavities made on the masticating surfaces. The amalgam (Dr. Bonwill's amalgam for fixing his crowns) is now mixed with sufficient mercury to make a firm though plastic mass, and, the cavities having been dried, a small portion of the mix is placed in and between the cavities, and worked in first with a burnisher, and then pressed home by placing on it pellet of bibulous paper, and applying to the pellet very considerable pressure with a blunt-pointed steel instrumen More amalgam is inserted and condensed in the same way and the process repeated until the cavities are complete filled, when, finally, great pressure is applied through a pellet of paper, by means of an instrument devised by Dr. Bonwill for fixing his artificial crowns (bicuspids and molars), which consists of an india-rubber buffer about half an inch in diameter, slightly projecting from a ferrule fixed to a wooden handle, the surface of the rubber and ferrule being at an angle of about forty-five degrees to the axis of the handle, to allow of its being applied to the back teeth. The fillings, or rather the filling—for at this stage there is only one—is now cut away until the bite is free (this being done first to prevent any chance of the amalgam being dislodged), and then roughly shaped at the cervical margin with thin steel instruments, but not divided at the surface, and the operation is completed for that sitting. On a subsequent day, when the amalgam has completely set, a division is made with a very thin band-saw, and the fillings shaped with hard rubber and corundum discs at the sides, and with conical fissure burrs at the cervical margins, and finally finished by polishing with pumice powder, &c.

"The principal peculiarities of this method are, filling proximal cavities together, by which much time is saved, and using considerable pressure on pellets of bibulous paper, by which process the amalgam is thoroughly adapted to the walls of the cavities, while all excess of mercury is squeezed out at the sides, and the crumbling—so common a source of trouble when using very dry amalgam—avoided.

"Dr. Bonwill frequently separates teeth by means of a temporary filling of red base plate, placed in and between the cavities, which, being bitten on, spreads laterally, and in the course of a month or so makes the requisite space."

Upon that method they had not further advanced in the making of water-tight amalgam fillings.

### DISCUSSION.

Dr. Elliott had heard some two years ago from Dr. Bonwill respecting his method of filling cavities with amalgam, and had filled cavities in that way with more or less success; but he certainly had not found the method to be superior to the old, as Mr. Tomes had done, from his experiments. He did not, however, consider that stoppings in patients' mouths could practically prove very much, as the conditions of each mouth varied so very much that it was impossible to get reliable information in that way.

Mr. Oakley Coles had only recently examined some specimens of amalgam sent out by Fletcher, of Warrington, some years ago, in glass tubes with Draper's ink underneath.

He had discovered that no leakage through had occurred, but the ink had penetrated the amalgam plug, clearly showing shrinking of the filling, to the extent of about two-thirds its depth. He considered a cylindrical form of cavity to be undoubtedly the best, and less liable to shrinkage than one which was shaped with a bevelled margin.

Mr. F. Canton said that gutta-percha fillings were not water-tight, and yet prevented decay. Was it necessary to have an amalgam filling water-tight to be a good one.

Mr. Stocken had always used his amalgams as dry as possible, so as to ensure a plastic stopping when finished. He thought that, by the Bonwill method of filling, the mercury squeezed out would carry with it some of the constituents of the amalgam.

Mr. S. Bennett said that Dr. Herbst had sent, early in the year, some specimens of amalgam plugs filled with rotary burnishers. Dr. Herbst laid down the rule that in preparing the cavities for his method of gold filling or for amalgam there must not be a chamfered edge of any description.

Dr. Field had not made any practical experiments with amalgams out of the mouth. He invariably used the rubber dam when putting in an amalgam filling, as he considered that better results could be obtained in that way.

Mr. W. H. Coffin was inclined to think very highly of the Bonwill method, for his very sufficient reason that his father had filled amalgam in the same way for the last fifteen or twenty years. Dr. Bonwill had undoubtedly introduced the method quite independently, and deserved every praise for bringing it before the profession.

Mr. Hutchinson, Mr. Wilson, and Mr. B. Mason joined in the discussion.

Mr. Tomes having briefly replied, the President called upon Dr. Field for his paper on "Pivot Crowns."

Dr. Field said the few remarks he intended to make had been suggested by what had been said at the previous meeting with reference to the durability of teeth pivoted by the old method. He had nothing new or original to bring forward on this subject, but he wished to offer one or two arguments in favor of a more modern or more elaborate method of attaching porcelain crowns to roots. He could bear witness to the durability of teeth pivoted by the old method, but he believed that at the present day dentists had to deal with very different conditions, and that their methods of practice must be modified accordingly. In the first place the quality of teeth which the practitioner of the present day had to work upon was not so good as that which fell to the lot of their seniors. In the second place it must be remembered that in former days it was rare that any but a sound root was pivoted, and that many teeth which would now be saved would then have been extracted without hesitation. The apparent success of the old method was not due to the means employed, but rather in spite of them, just as similar teeth were saved by gold and amalgam fillings inserted in a manner which would not be considered creditable at the present day.

It was with the inferior quality of teeth and under the less favorable conditions with which practitioners were now expected to deal, that he claimed superiority to the method he was about to describe, and which had been first introduced by the late Dr. Marshall Webb.

Supposing the root to be in a healthy condition, the first steps were to enlarge the canal to as near the apex as was prudent, close the apex, grind the end of the root level with the gum, and polish its marginal surfaces. A suitable crown must then be chosen, fitted to the root, and backed with thin gold. The pivot should be either square or triangular gold or platinum wire, sufficiently long to extend to the end of the canal when soldered to the backing. Next place the tooth and pivot *in situ*, fasten them together with wax, remove the whole carefully from the mouth, enclose in plaster, and solder the pivot to the backing. Then by means of corundum or emery discs ,cut a groove on both sides of the porcelain and across the cutting edge above the backing; into this the cohesive gold must be carefully packed. Having prepared the crown, adjust the rubber dam, fix the pivot in the root by means of a good quick-setting oxychloride cement, leaving a space of one or two lines between the porcelain face and the root. When the osteo is sufficiently hard, remove enough from around the pivot to obtain a firm foundation for the cohesive gold, then proceed to build up the contour of the tooth into the grooves already mentioned. When completed, finish with great care, giving special attention to the margins, this being the weakest point in all operations upon the teeth.

He had not gone into all the details of this method, but he thought the description he had given would enable anyone who chose to exercise his manipulative skill to fix such a tooth properly.

The advantages of the Morrison crown over a pivot tooth were, first, the ease with which they could be fitted to bicuspid and molar teeth; second, the firm support given to the root by the closely fitting band and the great strength of the whole combination; thirdly, they may be used on roots which would be condemned as useless for ordinary pivoting. The root should be prepared by cutting down to the gum, and removing with a fissure burr any inequalities of the periphery and all the enamel edges. A strip of 22-carat gold as wide as the length of the proposed crown is then fitted as accurately as possible to the root. The cap or cusps should then be struck up, fitted and soldered to the band, the depressions inside the cap having been previously filled up with solder. The root canals should be prepared as for the Webb pivot, and the wires fixed in the canals with cement in the same way; these should extend as far above the root as possible without coming in contact with the crown. The gum having been pressed from the root and the parts kept thoroughly dry, the crown is filled with cement, placed on the root and driven home with a few taps of the mallet; a small hole should first be drilled in one of the cusps through which the surplus cement may escape. Dr. Field then handed round one of the crowns, and also the steel dies from which they were made.

After some discussion, the meeting adjourned till November 2nd.—*The Dental Record.*

